# Developing a classification system and algorithm to track community-engaged research using IRB protocols at a large research university

**DOI:** 10.1017/cts.2021.877

**Published:** 2021-11-22

**Authors:** Emily B. Zimmerman, Sarah E. Raskin, Brian Ferrell, Alex H. Krist

**Affiliations:** 1 Center on Society and Health, Virginia Commonwealth University, Richmond, Virginia, USA; 2 L. Douglas Wilder School of Government and Public Affairs and Institute for Inclusion, Inquiry and Innovation - Oral Health Core, Virginia Commonwealth University, Richmond, Virginia, USA; 3 Center for Community Engagement and Impact, Virginia Commonwealth University, Richmond, Virginia, USA; 4 Department of Family Medicine and Population Health, Virginia Commonwealth University, Richmond, Virginia, USA

**Keywords:** Community engagement, natural language processing, tracking, classification, community-engaged research

## Abstract

Community-engaged research (CEnR) is now an established research approach. The current research seeks to pilot the systematic and automated identification and categorization of CEnR to facilitate longitudinal tracking using administrative data. We inductively analyzed and manually coded a sample of Institutional Review Board (IRB) protocols. Comparing the variety of partnered relationships in practice with established conceptual classification systems, we developed five categories of partnership: *Non-CEnR, Instrumental, Academic-led, Cooperative, and Reciprocal.* The coded protocols were used to train a deep-learning algorithm using natural language processing to categorize research. We compared the results to data from three questions added to the IRB application to identify whether studies had a community partner and the type of engagement planned. The preliminary results show that the algorithm is potentially more likely to categorize studies as CEnR compared to investigator-recorded data and to categorize studies at a higher level of engagement. With this approach, universities could use administrative data to inform strategic planning, address progress in meeting community needs, and coordinate efforts across programs and departments. As scholars and technical experts improve the algorithm’s accuracy, universities and research institutions could implement standardized reporting features to track broader trends and accomplishments.

## Background

The last few decades have seen increasing calls for universities to transform their relationships with communities and policymakers. By developing reciprocal relationships, universities seek to collaboratively produce knowledge with stakeholders, provide service that is beneficial to communities, and reaffirm a “*scholarship of engagement*” [1]. Many colleges and universities now identify community engagement as a core part of their mission [2]. Community engagement within universities cuts across teaching, research, and service missions [3]. The focus is on transformed scholarship that recognizes the value of lived experience and nonacademic expertise and the importance of partnership with organizations and communities.

Community-engaged research (CEnR) is a common component of the community engagement activities of universities and its benefits are increasingly acknowledged for the university as well as for diverse stakeholders and funders [4]. CEnR commonly includes patients, family members, health care providers, clinical researchers, community organizations, government entities, and other stakeholders [5]. A central focus of CEnR is to partner with underserved communities and population groups that are typically excluded from research. CEnR is conceptualized as a range of activities and commitments that indicate the depth and reciprocity of research team-community member relationships. Deriving from the International Association for Public Participation’s Spectrum of Public Participation [6], CEnR thought leaders theorize public participation in research as characteristics of “community involvement, impact, trust, and communication flow^”^ [7, p. 8] that deepen across, commonly, three to five levels of engagement

There are multiple reasons for measuring CEnR productivity and impact at the institutional level. Tracking can provide the data to plan for and support policy, infrastructure, and training needs [8], report to funders, and demonstrate accountability to the community and legislatures about scholarship that is responsive to community needs and incorporates community knowledge. Finally, this information can help to advance the field of CEnR generally. Despite the growing interest and investment, however, tools and metrics for tracking the type and volume of engagement are not widely available. Attempts to develop data-driven approaches specific to CEnR are complicated by the diversity of participatory research approaches, nomenclatures, stakeholders, roles, and research methods [9].

Unfortunately, the lack of efficient or automatable identification and tracking methods at the institutional level greatly limits information about the prevalence of CEnR and the types of engagement taking place. A survey of CTSA community engagement and evaluation program managers found that a number of metrics track typical return on investment (e.g., grants, training, publications), but metrics for engagement of stakeholders in research were uncommon [10]. To identify CEnR at one large mid-western university, investigators surveyed PIs of NIH-funded studies [11]. A survey of investigators was also conducted at Virginia Commonwealth University (VCU) in 2014 and 2019, but yielded response of only about 30%. While surveys can provide usable estimates, the data are cross-sectional, limited by nonresponse, and must be repeated, with significant effort, to be updated. To advance institutional metrics of CEnR, the current research seeks to systematize identification and classification to facilitate longitudinal tracking using available administrative data. The deep-learning approach takes advantage of natural language processing to categorize research by the level of engagement.

### Study Aims and Contributions

We describe an approach to identifying and tracking CEnR utilizing deep learning with data from human subjects protocols that have been submitted to the VCU Institutional Review Board (IRB). We describe the processes used to create a heuristic to categorize levels of community engagement, manually classify sample protocols to train an algorithm, and refine the algorithm to achieve reliable identification and classification of studies. We then present preliminary results and next steps.

Using IRB data and a deep learning algorithm to track CEnR promises several unique advantages. Using university-wide data resolves the problem of identifying research that is decentralized across many departments and settings. Natural language processing allows the algorithm to identify CEnR despite the distinct disciplinary approaches, terminology, methods, partners, and modes of engagement. Finally, the use of administrative data may be more reliable and easier to update than self-report surveys or database searches.

### Research Questions

Our study addressed three research questions about the volume and type of CEnR at VCU (a large, urban research university) and whether a deep learning approach could create reliable estimates and categorization of research.

- What is the annual volume of CEnR at VCU?
- What types of engagement characterize these studies?
- Can a deep learning algorithm accurately identify and classify CEnR using IRB protocols?

### Setting

This study was initiated at VCU in 2019 as a partnership between the Wright Center for Clinical and Translational Research (a CTSA hub) and the VCU Center for Community Engagement and Impact. VCU is a public research university and serves as an anchor institution for the Richmond, Virginia metropolitan area. It is designated an R1 Doctoral University with Very High Research Activity in the Carnegie Classification of Institutions of Higher Education.

**Fig. 1.**
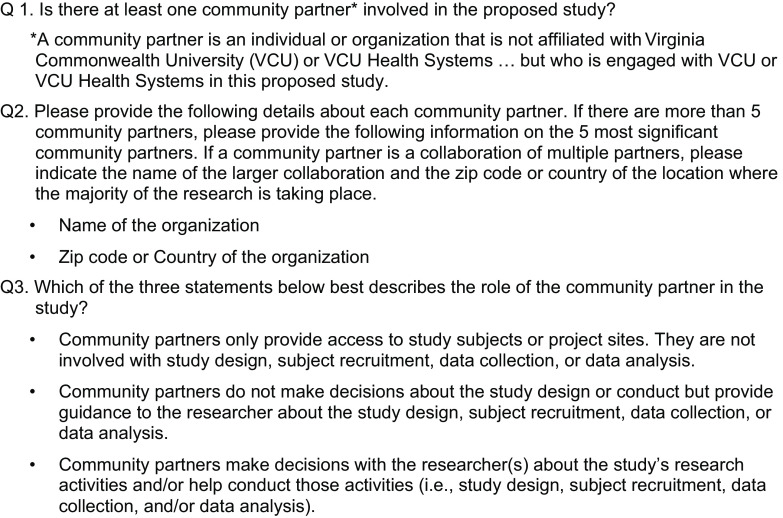
Custom Institutional Review Board (IRB) protocol fields.

The foundation for this study began in 2012, when a cross-university team sought to strengthen the documentation of CEnR at VCU. After evaluating options to track CEnR studies, including a faculty self-report survey and a GIS-based mapping approach, the team determined that IRB-based data collection would be the most systematic approach and developed a series of three questions that were added to the IRB application process in 2014 (see Fig. 1) [12,13]. The IRB process was selected because most research projects, funded and unfunded, are entered into the system. The questions asked if there was a community partner, who the partner was, and self-identification of the level of engagement. It was decided to only assess three levels of engagement – access to study participants, consultation on study design, or shared leadership. The project reported here is a next step for the IRB protocol tracking initiative. The multidisciplinary project team included community-engaged researchers from several disciplines (i.e., public health, social science, medicine) and a graduate student in computer science.

## Methods

### Creating the Dataset of IRB Records

We obtained human subjects protocols submitted between 2015 and 2019 from the online IRB protocol system (n > 6000). Preparation included removing incomplete and duplicate records and identifying fields of interest from the protocols (e.g., Background, Hypothesis, Study Design, Partner/Partnership Roles). After eliminating duplicates and records with null values in the selected fields, the dataset contained 2308 records.

To train the algorithm, we created an initial sample dataset that included protocols that indicated “Yes” or “No” to the question about involvement of a community partner (see Fig. 1). A random sample of 100 was selected using the random range button on Google Sheets. We added additional protocols (n = 180) using search terms for CEnR (community engaged, community based participatory research, (community) action research, participatory action research, community advisory group, community steering, community partner, etc.). The additional sample of 180 protocols was collected by uploading the full IRB data into an excel file and searching terms that related to the different categories of CEnR (as seen in the next section). These protocols were also randomly selected the same way.

### Developing Categories of CEnR

In the first round of coding the sample dataset, we focused on how the studies were classified based on the custom fields in the IRB protocol (Fig. 1). However, we found that the fields were better suited to tracking partnership arrangements than to identifying CEnR more generally. There was a wide variety of partners, including colleges and universities, schools, health care and service providers, community organizations, associations and coalitions, advocacy groups, pharmacies, treatment and recovery organizations, libraries, jails/correctional facilities, corporations, retail, housing, government agencies, arts organizations, faith organizations, practice-based research networks, community-university partnerships, research and technology organizations, and data repositories. In some protocols, the referenced community partner was actually part of the university system or a professional research partner. Additionally, we found instances of protocols where CEnR descriptors were elaborated in other fields, such as Research Procedures, but not logged in the three CEnR fields.

To proceed, we decided to inductively create classification levels based on the text found in the protocols. We also compared the protocol data with existing CEnR classifications to theorize the depth and mutuality of researcher-community partner relationship [6,14]. We noted that while these classification systems describe communication expectations, power-sharing arrangements, and other traits, they rarely connected the characteristics of CEnR studies to different types of research activities or problematized relationships commonly seen in higher education research settings, such as when a government agency contracts with a university to evaluate a program or when researchers access health system data from within the university hospital system or an affiliated clinic.

Using a ∼100 protocol subsample from the training sample of 280, we identified research activities that provided evidence of CEnR characteristics (e.g., partner organization facilitating access to data sources; community partner representatives reviewing study design, instruments, results, and dissemination plan; a community member being hired as research staff) as well as conventional indicators of CEnR such as specific research approaches (e.g., community based participatory research) or roles (community advisory board). We organized these characteristics into initial categories that reflected the practice of research, as described in human subjects protocols, and iterated them as we identified opportunities to clarify, differentiate, and strengthen the categories. We independently applied the categories (application by at least two researchers) to the sub-sample until we reached consensus on the categories. We resolved differences of categorization through discussion among the team members who manually classified the protocols. Once we reached consensus on the overall classification scheme, we applied it to the remaining ∼180 protocols in the sample. We again used discussion to reach consensus on any protocols that were more challenging to classify, often as a result of inadequate detail provided in the protocol.

### Algorithm Development

After comparing numerous algorithms with the potential for working well with textual data, we decided to use pretrained transformer-based models. These powerfully built language models are algorithms that learn from unlabeled datasets of text such as Wikipedia or BookCorpus (referred to as “unsupervised learning”) [15–17]. This process is important for the algorithm to build an understanding of how language is written mathematically so that it can be modeled for making predictions [18]. After being trained on large datasets, these models can be used for a second training task [19,20], such as our own classification task, which optimizes prediction-making [21] and minimizes data requirements. This form of algorithm development, referred to as “transfer learning” [22], uses algorithms trained on millions of parameters to jump start and brute force the learning process instead of depending on a single data source. Compared to traditional models trained on smaller datasets, these larger models can take better advantage of the context in which language is used [23]. This additional capacity is important for our model to delineate and generalize the patterns between the different levels of CEnR. This algorithmic learning style can, for example, recognize that words such as “community” or “engagement” can be written in a context that is outside of CEnR, whereas traditional deep learning techniques (e.g., GloVe Embeddings) would not be able to identify the different semantics of these words. Technical description of the training and evaluation of the algorithm will be published elsewhere.

## Results

### CEnR Classifications

Five distinct categories of CEnR emerged from inductively analyzing the protocol data, comparing analysis to existing classification systems to identify novel insights derived from a systematic analytic approach, and iterating the categories through application. Like other systems of CEnR classification, they reflect different levels of depth and mutuality in their typologies, from partnerships with the least engagement to those with the highest level of engagement, characterized by shared governance and reciprocity between the research team and the community members or partners. Adding to existing systems of classification, this set of categories derives from research protocol data and is intended to reflect the reality and limitations of CEnR.

1. ***Non-CEnR partnership***: This category references partnership within the protocol, but in ways that were otherwise uncategorizable due to limited evidence of specific engagement between the partners. This category also includes partnerships that are not generally considered community partners (e.g., external research entities, other universities or health systems, or commercial entities) and contractual relationships without collaboration.
  For VCU, this category provided a way to handle research characterized by contractual service relationships, as well as research by clinical faculty based in private practices and affiliated health systems. While traditional theories of CEnR have excluded such arrangements from classification as CEnR at all, the rise of clinician-investigator models in health care, the normalization of participatory action research approaches in education, and the acknowledgement of the diversity of stakeholders in community projects, including people in relative positions of power, provides the rationale for a category to contain such projects.
2. ***Instrumental partnership***: In this category, the community partner primarily facilitated researchers’ access to the “inputs” needed to conduct the study (e.g., posting recruitment flyers, providing participant contact information, extracting data, providing study sites for observation). Importantly, the partners included in this category are often important to the research due to their location, history, or reach in the community, but the partner may not have a specific stake or interest in the research topic. This category is analogous to categories in other systems of CEnR classification that refer to “outreach” or “informing,” with limited communication or involvement.
3. ***Academic-led partnership***: This category signals minimal yet important interaction between the research team and the community partner, which is often essential to project success. Studies in this category reflect shared interests between the research team and the community partner and an explicit commitment to using partnered research to advance community members’ well-being, but the level of engagement was “lighter touch” than categories higher up in the continuum. These studies generally operated under a traditional model in which academic partners take the lead on study design and research activities, with community partner involvement at particular points, such as troubleshooting recruitment or facilitating community meetings.
4. ***Cooperative partnership***: In this category, numerous activities in the research cycle were characterized by shared investment and mutual consideration between the research team and the community partner, but lacked shared decision making (e.g., community advisory boards that provided input on study design and methodology, reviewed data collection instruments, interpreted findings, informed dissemination plans). For some studies in this category, community members were hired as compensated project staff to recruit participants, collect data, and conduct other research activities.
5. ***Reciprocal partnership***: Studies in this category featured all of the characteristics of *cooperative partnerships* together with shared decision-making power and governance (e.g., community-based participatory research, team science, steering committees with decision-making power, participatory action research) between community partners and research teams.

### Making Predictions

As a first check on the preliminary results of the algorithm, we compared some of the manually coded protocols to predictions from the algorithm. Most of the sample that was manually coded could not be used for this purpose because it had been employed to train the algorithm, therefore a small subsample (n = 80) of protocols that were not used to train the algorithm was compared to the results. We used three categories where 0 = no partner (n = 17), 1 = Provides access to study subjects or project sites only (n = 27), 3 = provides guidance on the study or makes decisions with researchers (n = 36). There was an overall match rate of 73% between the manual codes and the algorithm codes. The highest match rate was for protocols rated 0 at 83% and the lowest was for protocols labeled 1 at 40%.

**Fig. 2.**
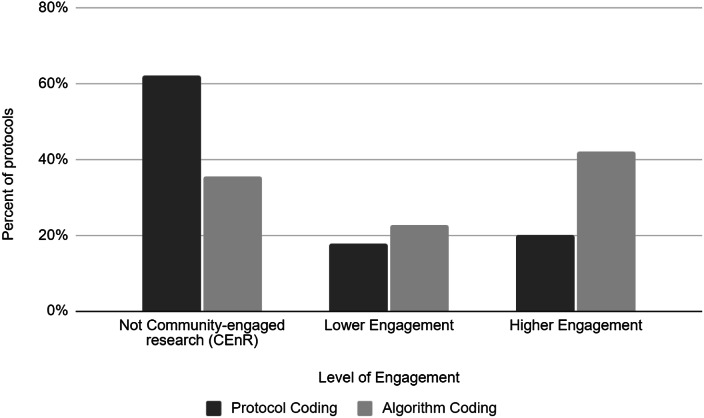
Comparison of investigator-reported partnership codes on Institutional Review Board protocols and algorithm coding. *Note*: “Not CEnR” includes code 0 from Institutional Review Board (IRB) partnership question (no partnership), and code 0 from the algorithm classifications (no partnership). “Lower Engagement” includes code 1 from the IRB partnership question (community partners only provide access to study subjects or project sites), and codes 1 (non-CEnR partnership) and 2 (instrumental partnership) from the algorithm classifications. “Higher Engagement” includes codes 2 (community partners do not make decisions about the study design or conduct but provide guidance to the researcher) and 3 (community partners make decisions with the researcher(s)) from the IRB partnership question, and codes 3 (academic-led partnership), 4 (cooperative partnership), and 5 (reciprocal partnership) from the algorithm classifications.

As a next step to assess results for all protocols in our sample, we compared the classification of the partnership questions included in the IRB protocol (Fig. 1) with the algorithm predictions for the CEnR classifications presented above. These preliminary results show that the algorithm is potentially more likely to categorize studies as CEnR compared to investigator-recorded codes indicating whether the study has a partnership (65% of protocols were coded by investigators as not CEnR, compared to 38% by the algorithm) (Fig. 2). In addition, the algorithm appears more likely to code studies at a higher level of engagement (42% vs 20%). Thus, our preliminary results indicate that the algorithm may be more sensitive to different types of engagement than the original set of IRB questions. One reason for this may be that the IRB questions required investigators to identify specific partners and did not focus on CEnR activities more generally.

## Discussion

### Implications

Currently there is a lack of systematic, automated, longitudinal, or comparative CEnR portfolio tracking tools within or across universities, which is a missed opportunity for reporting and planning purposes within institutions of higher education and other research entities. We set out to determine whether an algorithm could validate or improve on three questions embedded in IRB protocol fields. Our preliminary results indicate that the deep learning of the algorithm may be more sensitive to different types of engagement than the original set of IRB questions. One reason may be that the IRB questions required investigators to identify specific partners rather than CEnR activities or approaches. By training an algorithm that could detect diverse forms of engagement, the approach is more flexible in regard to terminology, partnering and collaboration arrangements, and approaches.

One of the strengths of the current approach is that it uses an asset that is already available to colleges and universities with online IRB systems, which are likely to be populated with the majority of studies. Our preliminary results demonstrate the potential for deep learning models/techniques to create efficient approaches toward assessing the full scope of CEnR across the university by automatically identifying, classifying, and longitudinally tracking CEnR using administrative data, with a focus on IRB protocols. A number of related uses are also possible, such as tracking the sustainability of CEnR efforts, examining partnership dynamics, and coordinating cross-university collaboration with community partners. The automated process can be used over time to assess trends and aggregate data across different schools and institutions. It could be applied to additional databases, potentially facilitating more coordinated cross-university collaboration with community members. Universities could use these data to inform strategic planning for their research portfolios, address progress in meeting community needs, and coordinate efforts across programs and departments. Moreover, scholarly and technical investment in the algorithm’s accuracy can position its introduction into more universities, which will permit CEnR data aggregation among external research and funding organizations. This will improve reporting on national trends and can inform future focus areas of CEnR.

The current approach also contributes to the development and refinement of an empirically based system of classification for CEnR studies. Through manually coding a sample of IRB protocols to train a deep-learning algorithm, we observed the variety of partnered relationships occurring at a large research university and developed a pragmatic continuum of CEnR that categorizes CEnR by considering characteristics of partners, their roles in the research process, and the nature of the relationship. This heuristic, we believe, provides a more nuanced classification of “levels of engagement” than continua that account for only one of these characteristics.

### Limitations

While our work represents a proof of concept that deep learning can be a useful way to identify and categorize CEnR using administrative data, we encountered several limitations related to categorizing CEnR, counting studies, and developing the algorithm. Technical barriers included lack of access to data entered as PDF uploads. This limitation would have resulted in an undercount of the total number of CEnR protocols at the institution but should not have had a substantial impact on the training or testing of the algorithm. In terms of algorithm development, we were limited in our ability to compute very large models due to limited GPU power. Finally, there are uncertainties in the process of developing the algorithm; the data and the output are observable, but how the algorithm derives the final result is not so transparent. The models will find the patterns to connect the inputs to outputs (e.g., discover the sequences of keywords, key phrases, add levels of attention of the focal point of a sentence, learn all the nuances of the reference categories), however, those features and decision criteria are not explicit.

A few limitations relate to the categorization function of the algorithm. The amount of detail about engagement that is contained in IRB protocols is often sparse, which could lead to misinterpretation of the level of engagement. For example, research projects with a collaborative research team – that is projects co-led by academic and community researchers – would qualify as CEnR but may not include language in the protocol that would be picked up by the algorithm if no organization was identified as a community partner. Often, these project protocols use terminology consistent with community-based participatory research, but that is not always the case.

Some limitations are specific to the use of IRB protocols, which provide information on one specific type of engagement (research involving human subjects) and therefore miss some components of CEnR (e.g., dissemination activities) and other types of engagement, such as service and learning. Undercounts could occur when investigators do not fully describe the engagement of stakeholders in their IRB protocol. Overall, the amount of error in detecting CEnR through IRB protocols that is due to self-report by investigators and variability in language and attention to different types of engagement in describing research protocols is unknown. And while engaged community partners may influence study design, it is unknown how often they contribute to the development of IRB submissions. Finally, our study focuses on specific research projects and does not assess research partnerships or partnership dynamics, which are critical to the success of CEnR.

### Future Research

Next steps for this work will focus on refinement and dissemination of the approach and use of the data within our institution. We plan to do extended comparisons of the data and publish detailed results of the algorithm’s prediction rate. Within the institution, we will work with VCU administration to incorporate the algorithm into ongoing assessment procedures to produce systematic longitudinal count and classification of CEnR, produce dynamic metrics for the VCU Center for Community Engagement and Impact website, and incorporate findings into reports to funders. We will identify opportunities to modify the IRB protocol system to better capture CEnR and provide guidance to faculty to describe participatory research in their IRB protocols. We hope to work with community partners and other colleges and universities to test and implement this method for identifying and categorizing CEnR.

Addressing the lack of systematic CEnR portfolio tracking using methods such as those described here may be of interest to research funders and research institutions. In addition to exploring other applications of using natural language processing to categorize research, future research could identify ways in which the evolving nature of CEnR influences the algorithm’s performance. Will intentional updating be required to capture changing approaches and nomenclature within CEnR? Will awareness of the tracking tool influence the ways in which investigators craft their IRB submissions? What are the implications of CEnR tracking and identification for strategic planning and partnership building?

## Conclusions

CEnR is now an established research approach, with a large, diverse body of studies. We developed a system for classifying CEnR studies at a research university that is home to a large number of disciplines in which CEnR is used. Derived by identifying key research activities and CEnR characteristics from IRB protocols and iterating the system of classification through dual manual coding and consensus, we created a pragmatic, empirically based, transdisciplinary CEnR classification system. Analyzing these studies in the aggregate provides evidence to amend earlier classification systems that rely on a simple continuum of engagement. Our classification scheme accounts for factors such as the type of partner, the type of relationship, and the level of engagement (e.g., cooperation vs shared decision making). The automatic process promises to be more accurate and less time consuming than investigator self-reports, allowing for more robust identification of CEnR across many siloes in a university. Longitudinal data will allow for assessing the evolution of and planning for CEnR. As scholars and technical experts improve the algorithm’s accuracy, national and global organizations could encourage universities to implement standardized reporting features to track broader trends and accomplishments and identify areas of CEnR that would benefit from focused attention, resources, or collaboration.
